# Measurements of Thermal Conductivity of LWC Cement Composites Using Simplified Laboratory Scale Method

**DOI:** 10.3390/ma14061351

**Published:** 2021-03-11

**Authors:** Marzena Kurpińska, Jarosław Karwacki, Artur Maurin, Marek Kin

**Affiliations:** 1 Faculty of Civil and Environmental Engineering, Gdansk University of Technology, Narutowicza 11/12 st., 80-233 Gdansk, Poland; marek.kin@pg.edu.pl; 2 Institute of Fluid-Flow Machinery, Polish Academy of Sciences, Heat Transfer Department, Fiszera 14 st., 80-231 Gdańsk, Poland; jkarwacki@imp.gda.pl; 3 Institute of Fluid-Flow Machinery, Polish Academy of Sciences, Hydropower Department, Fiszera 14 st., 80-231 Gdańsk, Poland; amaurin@imp.gda.pl

**Keywords:** thermal conductivity, measuring, simple laboratory, cement, lightweight aggregate, insulation, granulated ash aggregate

## Abstract

The implementation of low-energy construction includes aspects related to technological and material research regarding thermal insulation. New solutions are sought, firstly, to reduce heat losses and, secondly, to improve the environment conditions in isolated rooms. The effective heat resistance of insulating materials is inversely proportional to temperature and humidity. Cement composites filled with lightweight artificial aggregates may be a suitable material. Selecting a proper method for measuring the thermal conductivity of concrete is important to achieve accurate values for calculating the energy consumption of buildings. The steady state and transient methods are considered the two main thermal conductivity measurement approaches. Steady state is a constant heat transfer, whereby the temperature or heat flow is time independent. In the transient method, temperature changes over time. Most researchers have measured the conductivity of cement-based materials based on transient methods. The availability and cost of equipment, time for experimental measurements and measurement ability for moist specimens may be some of the reasons for using this method. However, considering the accuracy of the measurements, the steady state methods are more reliable, especially for testing dry materials. Four types of composites were investigated that differed in filler: natural aggregate, sintered fly ash filler, sintered clay and granular foam glass aggregate. The method of preparing the samples for testing is especially important for the obtained results. The samples, with a specific surface roughness, will show a lower coefficient of thermal conductivity by 20–30%; therefore, the selection of the type of contact layer between the plate of the measuring device and the sample is of particular importance.

## 1. Introduction

The effective thermal conductivity of cement-based materials such as concrete is an important factor when considering the amount of heat transfer through conduction. Many studies have demonstrated the impact of various parameters on the thermal performance of insulation materials. Extensive investigations have focused on heat transfer in these materials in the context of their numerous and diverse applications [1,2,3,4,5,6,7,8]. The amount of heat loss through walls has a direct effect on the energy consumption of buildings, which is why the thermal conductivity in these applications is one of the most important challenges facing thermal, mechanical, material, and civil engineers [9,10,11,12,13,14,15]. The thermal properties of composite materials such as concrete are affected by the thermal properties of each constituent material as well as the void space contained within the material. Factors influencing the thermal conductivity of concrete include age, aggregate volume fraction, amount of cement, types of admixtures, fine aggregate fraction, temperature, and moisture status. Moisture content, density, and temperature have been shown to most highly influence the thermal conductivity of concrete, along with aggregate volume fraction. Because concrete is typically comprised of aggregate for 60 to 70% (or more) by volume, the thermal properties of concrete are highly influenced by the thermal properties of the aggregates. A discussion of the thermal properties of a variety of natural aggregates, rocks, and minerals is presented by [16]. Porous materials have historically been used in building applications for their advantageous insulating performance. Various methods of measuring thermal insulation are used. The tests are carried out on samples of various sizes and surface roughness. The accuracy of measurement in the use of different techniques for evaluating the thermal conductivity of cement compo-sites is being discussed by scientists. [17,18,19,20,21,22,23,24,25,26,27,28]. It should be noted that several factors affect the thermal conductivity of concrete: the moisture content, temperature, density, porosity and adsorptivity of aggregate, and the properties of cementitious material are influential factors on the thermal conductivity of concrete [29,30,31,32,33]. Selecting a proper method for measuring the thermal conductivity of concrete is important to achieve accurate values for calculating the energy consumption of buildings. Various test methods are used to determine the thermal conductivity of materials, so it is difficult to say which method is most appropriate for this type of material. The steady state and transient methods are considered the two main thermal conductivity measurement approaches [34]. Recent research shows that steady state and transient heat transfer are considered different heat transfer conditions across materials. Steady state is a constant heat transfer, whereby the temperature or heat flow is time independent. The transient method is dependent on time and the temperature changes over time. Researchers have measured the conductivity of cement-based materials with transient methods, and results were presented in [35]. The availability and cost of equipment, time for experimental measurements and measurement ability for moist specimens may be some of the reasons for using this method. However, taking into account the accuracy of the measurements, the steady state methods are more reliable, especially for testing dry materials [36]. The purpose of the conducted research is determination of the influence of the surface quality and humidity of the cement composite on the assessment of thermal conductivity.

Due to the known influence of materials porosity on insulation performance, the authors offten predictive models for the thermal insulation performance of concrete taking into account the density, which in turn is closely related to porosity. The authors presented a simple exponential relationship between density, porosity and thermal conductivity in works [37,38]. A two-phase theoretical model for the prediction of the thermal conductivity of concrete was developed by [39] using the mortar and aggregate as the two components of the model. Similar two-phase and multi-phase models for the thermal conductivity of porous materials are referenced in work [40], and an approach considering an idealized system of “open and enclosed pores” has been proposed by [41]. Other researchers have developed trained neural network models to predict the thermal conductivity of concrete, although measured values were not used to train the models to evaluate the algorithms [42]. Several publications provide results of empirical studies of concrete’s thermal conduc-tivity and heat capacity, typically using concrete or mortar made with natural aggregates. The authors showed that thermal conductivity depends on a number of factors including age samples, water-cement ratio (w/c), types of ad-mixtures, type of aggregates and the humidity of the specimen [43,44]. In work [45], researchers presented the results of the performed study on the thermal conductivity of ternary mixtures (using three types of coarse aggregates and natural sand), varying moisture content to explore the sensitivity of test results to moisture state. In [46], a summary of heat capacity results from these tests was provided. A linear decrease in heat capacity with an increasing moisture content and a significant effect from change in aggregate type were shown. For each of these studies [43,45,46], models were developed, but lightweight aggregates (LWAs) were not included in the experimental program, while The authors [47] developed models for lightweight concrete (LWC) taking into account using pumice and expanded perlite aggregate. The testing was performed using the standard methods [48], where specimens are not tested at steady-state. From the results, a model with strong correlation was developed between in an air-dry condition state specimens and thermal conductivity at specimens after oven-dried in temperature 105 C. In [49] are presented study to evaluate the specific heat of selected concretes with density of 640 to 2338 kg/m^3^ was performed. Thermal conductivity of the LWC and NWC were determined experimentally in the saturated condition. The authors found that aggregate type (and hence, concrete unit weight), as well as moisture content, strongly influenced heat capacity [49].

This study focuses on the creation of a test stand for measuring the effective thermal conductivity of lightweight concretes in particular. The hot plate method was chosen due to its great accuracy.Generally in this case, the test samples are fixed between hot and cold plates. In this method, measurement parameters are recorded as a tested materials thermal state reaches complete equilibrium. It means that the temperature at each point of the specimen is constant and the temperature does not change with time. A constant heat stream flows over the test samples. Thermal conductivity is determined through the heat flow rate and the difference in mean temperature between the specimen surfaces. Detailed instructions on the construction of a measuring instrument for such measurement can be found, for example, in the relevant industry standards [50,51]. The presented design solutions ensure the achievement of the defined, high measurement accuracy. A similar level of measurement accuracy can be achieved using simplified solutions, as presented in this paper.

## 2. Materials and Methods

### 2.1. Materials Characterization

All mixtures used a portland cement CEM I 42.5R according to [52,53]. Properties of cement are presented in Table 1 and Table 2.

**Figure 1:**
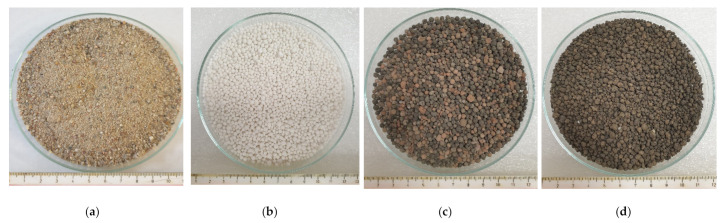
Aggregates used in research. (**a**) natural aggregate (NA) 0–4 mm; (**b**) foamed glass aggregate (GA) 0–4 mm; (**c**) expanded clay aggregate (CA) 0–4 mm; (**d**) granulated fly ash aggregate (AA) 0–4 mm.

The following aggregates were used for the tests: natural aggregate 0–4 mm (NA), foamed glass 0–4 mm (GA), expanded clay 0–4 mm (CA) and sintered fly ash 0–4 mm (AA). The aggregates meet the requirements of standards [54,55]. Properties of aggregates are presented in Table 3. The aggregates are shown in Figure 1.

### 2.2. Mix Proportion and Mixtures

The research program included the testing of 4 concrete mixtures. One mix containing natural fine aggregate was the reference mixture, and the other three mixtures contained light aggregate in various proportions.

The concretes were prepared in two stages: Firstly, a cement matrix in the form of mortar of a specified nominal water–cement ratio was made, and was then added to a lightweight aggregate of a specified moisture content condition in a certain amount to allow a workable consistency of S3 to be reached; this was determined according to Ref. [55]. Therefore, the proportions of fine aggregate and cement matrix were changeable for lightweight and natural fine aggregate series and were mainly determined by the initial moisture content, which affected the capacity of the aggregate to open porosity and adsorption water from fresh concrete. The mixtures prepared with aggregates of a higher adsorption and/or were characterized by a greater share of cement matrix in their compositions. The ratio volume of fine aggregate was constant for all mixtures and was 490 dm^3^. The parameters and compositions of prepared concretes are given in Table 4 and Table 6.

Due to their substantial water adsorption, prior to their application in the concrete mix, the lightweight aggregates were preliminarily water saturated within 24 h.

All mixtures had the same water/cement ratio of 0.44. The aim of the laboratory tests was to determine the influence of the test method, sample preparation method, their porosity and moisture content on the study of the thermal conductivity coefficient of normal and lightweight concrete. The composition of concrete mixtures is shown in Table 4.

The components of the mixture were mixed in a mechanical mixer. First, cement and water were mixed for 2 min. Then, the aggregate was added to the cement paste in accordance with the designed composition of TC NA, TC GA, TC CA and TC AA and mixed for another 2 min. Consistency was tested after 15 min by means of the slump method in accordance with [56]. The consistency of all the mixtures in accordance was within the range of S3 (100–150 mm) [57]. To make samples for compressive strength testing, the concrete mixture was laid in PVC molds of the size 15 × 15 × 15 cm^3^ in two layers and vibrated on a vibration table in accordance with [58]. Of each type of concrete, three samples of the size 15 × 15 × 15 cm^3^ were prepared to determine the density of concrete in accordance with means in compliance [59].

The specimens for testing the thermal conductivity coefficient were made in special forms of 30 × 30 × 5 cm^3^. The form was filled vertically in such a way that the surface of the plate, 5 cm thick, was very even and smooth. The form was filled and compacted in 3 layers in order to remove air from the concrete mix.

The summary of the size and quantity of the specimens for testing is presented in Table 5.

**Figure 2:**
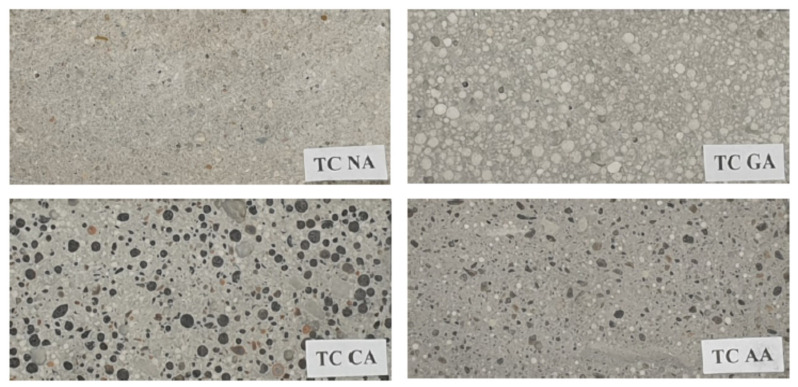
Sample structures.

All test specimens were stored for 24 h in their molds, at a temperature of 20 ± 2 °C, followed by subsequent storage in the chamber with the humidity of 95–100% and the temperature of 20 ± 2 °C and protected against drying. Sample structures are shown in the photographs in Figure 2.

### 2.3. Research

Density concrete test was conducted by means of the volumetric method. Before the start of the tests, three specimens of each variant of TCNA, TCGA, TCCA, and TCAA had been dried at the temperature of 105 °C until they reached a steady mass state according to [59]. Each result is an arithmetic mean of three independent mass measurements. Volume density was calculated on the basis of the following pattern:

(1) $$\rho =\frac{m}{V},$$

where: *ρ*—sample’s volume density, [g/cm^3^]; *m*—mass of the specimen, dried at the temperature of 105 °C [g]; *V*—volume of the specimen, [cm^3^].

Compressive strength of concrete was tested at 28 days by means of the Controls Advantest 9 (Controls, San Maurizio Canavese, Italy) machine of a maximum pressure force of 3000 kN in accordance with [59,60]. Compressive strength is an average value of six results obtained.

The specimens to the first stage of the thermal conductivity coefficient test were in a wet state. Next, the samples were placed in an oven and dried at the temperature of 105 °C to constant weight (for 4 days). After drying, the thermal conductivity coefficient test was repeated. Before testing, the dimensions and weight of the sample-plates were determined. The surfaces of all the specimens were properly finished to achieve smooth surfaces in order to maintain the proper contact between the cold/hot plate and the specimen.

Thermal conductivity coefficient *λ* was calculated by means of inner and outer model enclosure temperature measurement, and also by measurement of the inner and outer wall temperature of the tested specimen. The temperature was measured every 10 min since establishing a balance state on the sides of the specimen. Thermal balance on the walls of the specimen was achieved after 5–6 h, but to receive more detailed test results, the temperature was measured for 8 h every 10 min. The following results of the measurements were registered: *T_Li_*—chamber inner temperature [°C]; *T_wi_*—specimen inner temperature [°C]; *T_wa_*—specimen outer temperature [°C]; *T_La_*—temperature of the model enclosure surrounding [°C].

**Figure 3:**
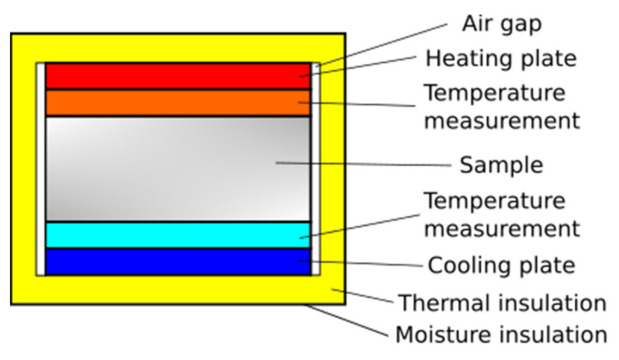
Calorimeter schematic diagram.

Thermal conductivity was determined through the heat flow rate and the difference in mean temperature between the specimen surfaces. Detailed instructions on the construction of a measuring instrument for such measurement can be found, for example, in the relevant industry standards [50,51]. The presented design solutions ensure the achievement of the defined, high measurement accuracy. A similar level of measurement accuracy can be achieved using simplified solutions, as presented in this paper. The layout of the experimental instrument is shown in Figure 3.

**Figure 4:**
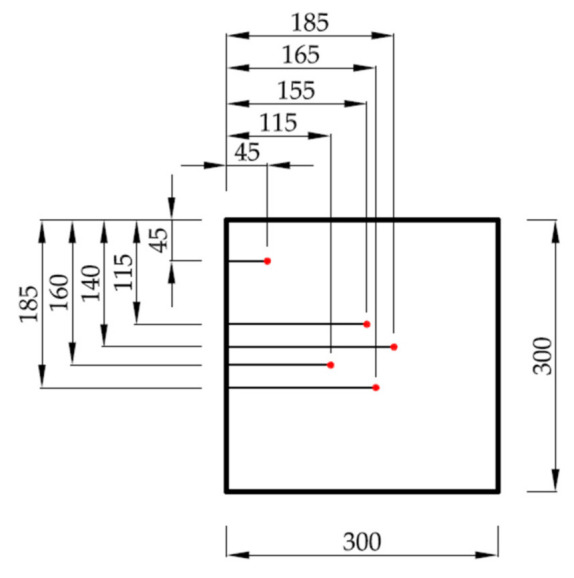
The distribution thermocouple rows on temperature measurement plate (dimensions are shown in millimeters). The red dots indicate the temperature measurement points.

**Figure 5:**
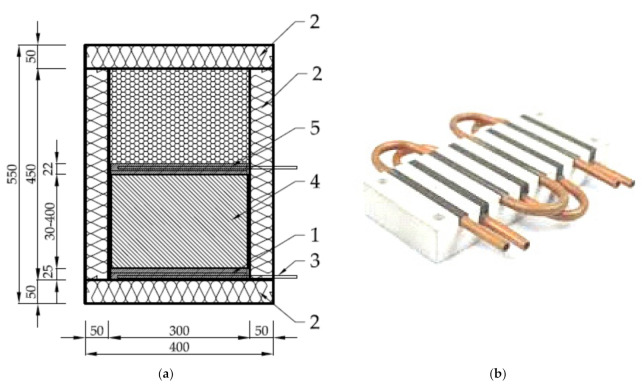
Main dimensions in millimeters of the calorimeter (**a**): 1—cooling and low temperature measurement plates, 2—thermal and moisture insulation, 3—cooling coils, 4—sample material, 5—heating and high temperature measurement plates. Photo illustrates the cooling coils arrangement method (**b**).

The sample is covered from its top and bottom by temperature measurement plates. The plates, made from PA13 aluminum alloy (300 mm × 300 mm × 10 mm), have five grooves (1.5 mm × 1.5 mm) for conducting k-type thermocouples (Figure 4), placed on the sample sided (inner) surface. Four of the thermocouples’ heads are placed evenly near the center, and the fifth one, for temperature distribution reference, is placed in the corner of the measurement plate. The heating and cooling plates are adjacent to both measurement plates. The heating plate is made from PA13 aluminum alloy (300 mm × 300 mm × 10 mm) with an attached 350 W, 24 V surface electric heater. The cooling plate, made from the same material, has the dimensions 15 mm × 300 mm × 300 mm. The higher thickness compensates the depth of twelve parallel coil grooves (5 mm × 5 mm) carved though the whole surface length. The photograph in Figure 5 illustrates the f 5 mm cooling coil arrangement in those grooves. As a whole, the above mentioned components constitute a measurement stack.

Outside the measurement stack, thermal and moisture insulation are provided in the form of an air tight box from styrofoam. A 4 mm air gap between the insulation’s inner surface and the sample side walls provides additional thermal insulation. The insulation box can disassemble to change the sample. The overall dimensions of the calorimeter as well as the cooling coil arrangement method are presented in Figure 5.

**Figure 6:**
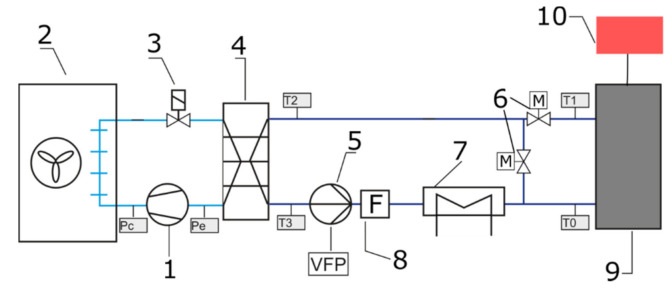
Layout of experimental setup: 1—compressor, 2—condenser, 3—electronic expansion valve, 4—evaporator, 5—circulating pump, 6—control valves, 7—electric heater, 8—flowmeters, 9—calorimeter, 10—electric heater power supply.

An experimental setup was designed and built to achieve stable measuring conditions. The layout of the experimental setup is shown in Figure 6. Two individual loops, separated by the heat exchanger **4**, can be identified. The first loop (left of Figure 5) is a typical compressor refrigeration cycle. To achieve more stable working conditions, a commonly used thermostatic expansion valve was replaced by electronic device **3**. Our own algorithm of valve control allowed us to reduce working parameter fluctuation. During the experiment, the refrigerant parameters were calculated based on the CoolProp library [33]. In the second loop, water was used as the heat transfer fluid. To achieve fast temperature response of the system, the amount of water was minimized. Water chilled in the evaporator is pumped to the cold plate **9** by the variable speed circulating pump **5**. A heat transfer fluid flow rate was kept constant by regulating the rotating speed of the circulating pump. The flow rate in all cases was set to 120 kg/h. To maintain the set inlet temperature of water, electric heater **7** was used. To minimize the fluctuations of inlet temperature, the electric heater was equipped with the precise single-phase power controller JUMO TYA-201 (JUMO GmbH, Fulda, Germany). Coriolis flow meters **8** were used to measure the heat transfer fluid mass flow rate. E&H Promass 83F DN08 mass flow meters (Endress+Hauser AG, Reinach, Switzerland) with an accuracy of 0.3% were used in the measurements. A high accuracy power supply **10** (Twintex PPS-3030, Twintex Electronics Co., Ltd., New Taipei City, Taiwan) was used to power the electric heater. To set and read supply parameters, the RS-232 interface was used. The applied control systems allowed for high precision of experimental conditions.

**Figure 7:**
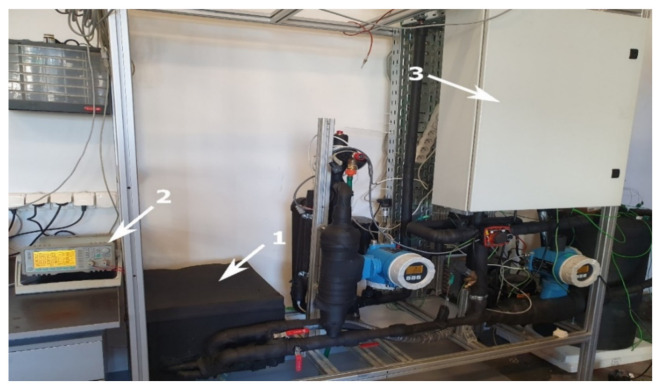
Photo of the experimental stand: 1—calorimeter, 2—electric heater power supply, 3—control and measurement system.

In the key points of the experimental stand, K type thermocouple sensors were installed. Two temperature sensors were located at the inlet T0 and outlet T1 of the cold plate. To achieve a fast temperature response, the thermocouple K-type sensors with 1 mm shield diameter were used. All thermocouples were calibrated and tested before installation using the Beamex MC6 calibrator (BEAMEX OY AB, Pietarsaari, Finland), the Druck DB-150 calibration furnace (GE-Sensing, Billerica, MA, USA) and the reference temperature sensor PT100 Isotech (Isothermal Technology Ltd., Southport, UK). After this process, the precision of the thermocouples was ±0.1 °C for 25 °C. The photo of the experimental setup is shown in Figure 7. The box with the specimen is visible in the foreground on the left side.

Each of the experiments carried out lasted for at least 12 h. For this reason, the real-time controller was used for measurements to obtain the required experiment parameters. The data acquisition facility was based on National Instrument systems and software. The NI cRIO 9014 controller (NI, Austin, TX, USA) was used as a main part of the data acquisition and regulation system. This system logs all main parameters and controls the valves, pumps, electric heaters, power supply and safety system. During the experiments, the real time measurements are shown on the computer screen while all measured data are stored in a data file.

## 3. Data Reduction

Detailed guidelines for the accurate measurement of the thermal resistance of specimens are given, for example, in EN 12664 [50] (products with medium and low thermal resistance) and in EN 12667 [51] (products with high and medium thermal resistance, no less than 0.5 m^2^·K/W thread). The test stand described in the study, and in particular the measuring apparatus, differ significantly from those presented in the above standards. Due to the significant complexity of normative constructions, a simpler design was made (Figure 3). This type of solution is also often used in research works [61,62]. The applied method and the geometry of the apparatus force a special emphasis on the analysis of measurement uncertainty and the minimization of all factors negatively influencing the accuracy of measurements. In many works using steady state methods, the analysis of measurement errors is omitted. The discussion about the preparation of the sample surface for such measurements is also omitted. Without the measurement uncertainty, it is difficult to determine whether the obtained values are reliable. These issues are essential when measuring the heat conductivity coefficient. For this reason, their detailed discussion in relation to the presented test stand and measurement method is presented below.

The calculations of the thermal conductivity were based on the following assumptions:

- Heat transfer along the radial direction of the specimen was neglected;
- The specimen was considered as homogeneous.

Based on the above assumptions, the temperature distribution in the specimen can be expressed through the one-dimensional Fourier’s Law:

(2) $$q=-\lambda \frac{dT}{dx},$$

where *x* is the dimension along the heat conduction direction. Assuming that we know the surface temperature of the specimen, the thermal conductivity can be expressed by the following relationship:

(3) $$\lambda =\frac{\dot{Qd}}{A\left(T_{h}-T_{c}\right)},$$

where *T_h_* and *T_c_* are the hot and cold specimen surface, respectively.

The assumptions made above are met only to a limited extent in real life conditions. Divergence with them causes, in some cases, huge measurement errors.

Preliminary tests were conducted using a concrete specimen from a typical mixture used for paving slabs (not included in Table 1). For these tests, it was assumed that the temperature of the cooling surface would be kept at 10 °C and the heating surface at 30 °C. Such test conditions were assumed to be more similar to the natural conditions of lightweight concrete application. However, the measuring apparatus did not provide full protection against the penetration of moisture into the measuring area. For a room humidity of 50% and an air temperature of 20 °C, the dew point temperature is 10.7 °C, so condensation may occur on the cooling plate as well as on a part of the tested sample. The heat resulting from the condensation process is not taken into account when calculating the thermal conductivity and can cause significant measurement errors. In addition, the liquefaction process continuously changes the conditions of the experiment. The condensate, which fills the space between the cooling plate and the specimen very slowly, significantly changes the contact resistance between these surfaces. These changes are so slow that the measurement in this case can be erroneously considered as a steady state. Within a few hours, the measurement conditions change and the electrical power supplied to the hotplate also changes. Therefore, significantly different values of the resulting heat penetration coefficient are obtained. In order to avoid the described effect, the temperature settings have been changed to 20 °C for the cooling plate and 40 °C for the heating plate, respectively. Adopting such measurement conditions protects the device against moisture migration inwards due to the fact that the average air temperature around the sample is 30 °C.

Another important issue in the measurement process is to estimate or eliminate heat loss to the environment. For the assumed conditions of conducting the experiment, 20 °C and 40 °C, respectively, and an ambient temperature of 20 °C, the average temperature difference between the inside and outside of the apparatus is 10 °C The roughly estimated heat loss to the environment for one calorimeter wall is 0.48 W. This means that for the whole apparatus the estimated heat loss is about 3 W. This value is significant because for lightweight concretes with a heat conduction coefficient below 0.5 W/m·K, the electrical power of the heating panel is between 10 and 20 W. For this reason, the ambient heat loss is a significant component of the measurement error. These losses must be estimated or measured and then taken into account when calculating the heat conduction coefficient.

**Figure 8:**
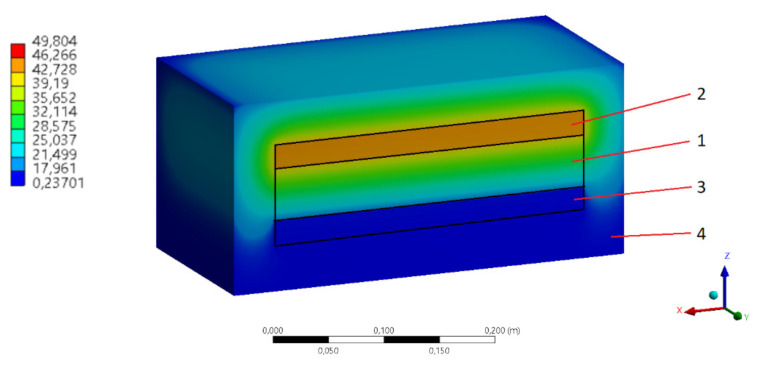
Temperature distribution in the calorimeter FE model (cross section): 1—sample, 2—heating and high temperature measurement plates, 3—cooling and low temperature measurement plates, 4—thermal and moisture insulation.

Due to the design of the apparatus, a simple estimation of heat loss to the environment may be too imprecise. Therefore, additional numerical calculations were made. Steady state thermal computational analysis was performed in the ANSYS 2020R1. The calorimeter structure is simplified in a three-dimensional model, as shown in Figure 8. The cooling and the heating plates were integrated with their measurement plates. The low temperature boundary condition (20 °C) is provided on the bottom surface of the cooling plate. The high temperature boundary condition (40 °C) is provided by a heater integrated on the top of the heating plate. The x-axis and y-axis are parallel and the z-axis indicates the direction of the main heat flux. The outer dimensions of the calorimeter are ~100 × 300 × 300 mm. The ambient temperature is 22 °C. The model has over 470,000 nodes and 188,000 tetrahedral elements. The material properties used in the simulation were implemented directly from the ANSYS 2020R1 material library: to calibrate the device the sample was made from quartz glass, the heating and cooling plates were made from aluminum, and the thermal and moisture insulation were made from polyurethane foam.

The temperature distribution in the apparatus is shown in Figure 8. The total heat losses to the environment calculated in this way are greater than those previously presented and amount to 5.1 W. Such a value was taken into account for all measurements made. As can be seen in this case, for materials with high thermal resistance, ambient heat losses could be the most significant component of the measurement error.

The experimental stand is dedicated to testing samples of concrete mixtures with a unique composition. For this reason, it is often impossible to obtain a set of the specimen with the same parameters. Due to the research character of the test stand, we decided to build the measuring apparatus in a single-sample system.

In the case of different concrete mixtures, we also deal with different sizes of aggregate grains. The process of sample preparation itself also influences the formation of typical areas with air bubbles, which after solidification form pores of different shape and size. The selection of the sample size should be adjusted to the size of grains or pores in the material. Of course, such material can only be regarded as homogeneous. In this case, heat exchange not only takes place by conduction, but also, in the pore area, by convection and radiation. For this reason, the concept of effective heat transfer coefficient is often used.

The size of the sample should be selected so as to cover the entire surface of the heating module. The differences in the linear dimension should not be greater than 3%, which for a 300 mm sample is 9 mm. The sample thickness should be greater than 10 times the average size of grains, granulate or other components added to the mixture.

A high degree of flatness and smoothness of the sample surface in contact with the heating and cooling plate shall be ensured so that close contact between the samples and the sample plates is possible. If necessary, the faces of the sample should be flat ground. Preliminary testing shows that the incorrect preparation of the contact surface can cause measurement errors of several dozen percent. In the case of contact surfaces with high roughness, for which the grinding process cannot be carried out, conductive pastes can be used to reduce the effect of contact resistance on the measurement result. This solution is used quite often [63]. As part of the test stand testing, tests were carried out using such a paste. This has made it possible to significantly eliminate contact resistance and increase the accuracy of thermal resistance measurement. However, the inconveniences associated with the application of such a solution indicate that a better approach is to prepare the contact surface accurately, e.g., by grinding. Another solution is to install special contact sheets made of compressible material between the surface of the sample and the heating/cooling element of the apparatus. Such sheets allow the establishment of good thermal contact between the surfaces and therefore should have the lowest possible thermal resistance [50]. Unfortunately, the use of contact sheets forces the temperature sensors to be placed directly on the surface of the tested sample, which significantly complicates the process of sample assembly in the apparatus. For this reason, the adopted solution assumes placing the temperature sensors in the heating and cooling plate as close to the surface of the specimen as possible.

During the experiment, the recording of measurement data was performed continuously. The data were recorded in the controller’s non-volatile memory. Parameters were recorded 2 times per second and averaged values from 10 samples were saved to the data file, i.e., the recording took place every 5 s. In this way, 1000 measurements were saved to one file. Dedicated software was created in LabVIEW environment to process the measurement data. The data read from the file were averaged again after 60 samples, which corresponds to 5 min of measurement (60 × 5 s = 300 s). For each of the 60 samples selected in this way, linear interpolation was performed and the directional factor was determined. The measurement with the lowest value of the directional factor was assumed to be appropriate, which corresponds to the steady state conditions.

## 4. Results and Discussion

For the thermal conductivity test, specimens of size 30 cm × 30 cm × 5 cm were used. The specimen plates were dried in the oven for 4 days at the temperature of 105 °C. The thermal conductivity of the three specimens (TC GA, TC CA, TC AA) was compared with the conventional material (TC NA). The obtained measurement results are presented in Table 6. The table includes:

- The density of the dry specimen measured directly after the measurement of the heat conductivity coefficient,
- The average heat conductivity coefficient;
- The maximum measurement uncertainty of the average heat conductivity coefficient;
- The percentage measurement uncertainty of the average heat conductivity coefficient;
- The heat resistance of the specimen.

The uncertainty of measurement has been calculated for the following uncertainties of measurement of the individual components:

- Cooling plate average temperature 0.16 °C;
- Heating plate average temperature 0.16 °C;
- Electric power 0.02 W;
- Specimen thickness 1 cm;
- Specimen surface area 2 × 10^−6^ m.

On the basis of the results of laboratory tests, it can be concluded that the kind aggregate has an enormous influence on concrete properties. Namely, grain porosity and resistance to crushing significantly influence the density, porosity, compressive strength and thermal conductivity of concrete.

The content of the given aggregate in cement composites can be considered a macroscopic parameter, defining the impact of physical properties of the aggregate on the properties of the composite. The aggregates, applied in research, can be described using such parameters as the presence of the empty spaces inside or outside grain of them with or without moisture environment, and the impact of the aggregates on the composite’s porosity.

The above mentioned parameters can be considered microscopic parameters of local layouts between grain and cement paste. The compressive strength of concrete is advantageously influenced by NA, which is of higher strength and lower porosity. In tests, the highest average density TC NA was 2244.4 [kg/m^3^] and the compressive strength TC NA was 67.5 [MPa] where natural aggregate was used. In case the same volume ratios of lightweight aggregate, if used foam glass aggregate the results were significantly different and average density TC GA was 45% lower while and compressive strength was 32% lower than TC NA. If used expanded clay aggregate, average density TC CA was 32% lower while compressive strength was 15% lower than TC NA, and if used granulated fly ash aggregate instead natural aggregate, average density TC AA was 28% lower and compressive strength was only 7% lower than TC NA.

**Figure 9:**
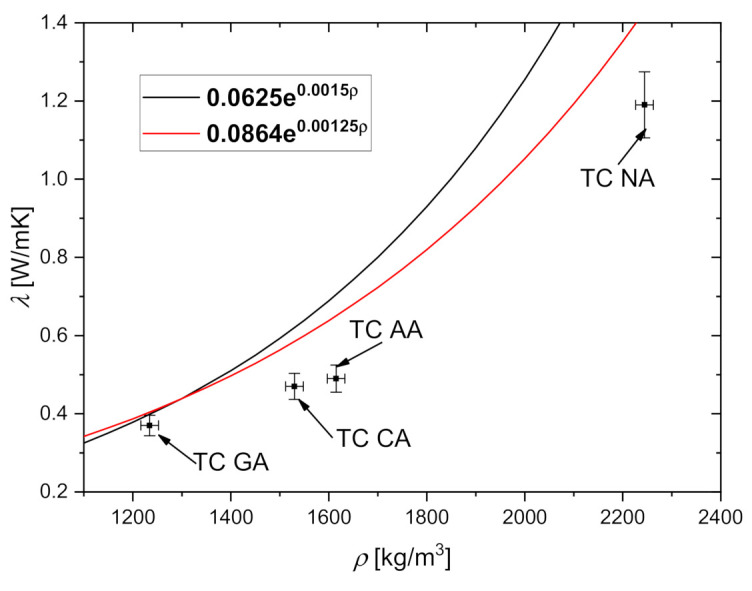
Relationship between thermal conductivity of the specimens. For comparison, predictions of Equations (4) and (5) are also shown.

The thermal conductivity parameters in function of density TC NA, TC GA, TC CA and TC AA are presented on Figure 9. 

In tests, the highest average thermal conductivity showed concrete witch natural aggregate TC NA and was 1.19 [W/m·K] and the thermal resistance TC NA was only 0.042 [m^2^·K/W]. If used foam glass aggregate the results were different and average thermal conductivity of TC GA was 69% lower while and thermal resistance was until 221% higher than TC NA. If used expanded clay aggregate, average thermal conductivity of TC CA was 60% lower while thermal resistance was 106% higher than TC NA, and if used granulated fly ash aggregate instead natural aggregate, average thermal conductivity of TC AA was 59% lower and thermal resistance was 102% higher than TC NA.

(4) $$\lambda =0.0625e^{0.0015\rho },$$

(5) $$\lambda =0.0864e^{0.00125\rho },$$

Equation (4) was derived from 185 experimental data points [35]. This relationship is presented as a black line in Figure 9. As can be seen, there is a growing discrepancy between the obtained results and Equation (4) as the density increases. However, this relationship was obtained for a wide range of concretes and its correlation coefficient is not very good. The other correlation, Equation (5), is recommended by the American Concrete Institute (the red line in Figure 9). The equation proposed by the ACI committee [35] is recommended for non-structural lightweight concretes and seems to reflect the present results with satisfactory accuracy.

## 5. Conclusions

The purpose of this paper was to selecting a proper method for measuring the thermal conductivity of concrete is important to achieve accurate values for calculating the thermal resistance of materials. The steady state and transient methods are considered the two main thermal conductivity measurement approaches. Steady state is a constant heat transfer, whereby the temperature or heat flow is time independent. In the transient method, temperature changes over time. The physical properties: density, compressive strength and thermal conductivity of four different concretes was tested. Was used natural and three artificial aggregates. Considering mechanical properties, the lightweight aggregate is the weakest component in concrete, which have a great influence on the compressive strength of cement composites. Nevertheless, analyzing four different concretes due to other physical properties such as density and thermal conductivity, it was revealed that the material offers multiple advantages. 

The use of porous aggregate made of foamed glass can reduce the weight of concrete with natural aggregate by as much as 45% with a decrease in strength by 32%, but the thermal resistance in this case increases by as much as 221%.

Otherwise, if we use expanded clay instead of natural aggregate, the decrease in strength compared to ordinary concrete will be at the level of 15%, the weight loss by 32% and the thermal conductivity resistance will increase by over 150%. However, if we use artificial aggregate with relatively high strength, e.g., granulated fly ash, the compressive strength will drop by only 7%, the weight will decrease by approx. 28%, while the heat conduction resistance will increase by over 140%.

Selecting a proper method for measuring the thermal conductivity of concrete is important to achieve accurate values for calculating the energy consumption of buildings. The steady state and transient methods are considered the two main thermal conductivity measurement approaches, specifying different heat transfer conditions across materials. In research, a simple in assumption measurement method was used to determine the heat conductivity coefficient. In this type of measurement, special attention should be paid to factors that can increase the measurement error. These are primarily heat loss to the environment and contact resistance. Despite many disadvantages, this method of measurement provides reliable and accurate results. The availability and cost of equipment, time for experimental measurements and measurement ability for moist specimens may be some of the reasons for using this method. However, considering the accuracy of the measurements, the steady state methods are more reliable, especially for testing dry materials. Four types of composites were investigated that differed in natural aggregate filler, sintered fly ash filler, sintered clay and granular foam glass aggregate. The method of preparing the samples for testing is especially important for the obtained results. Samples, but with a specific surface roughness, will show a lower coefficient of thermal conductivity by 20–30%; therefore, the selection of the type of contact layer between the plate of the measuring device and the sample is of particular importance.

## Figures and Tables

**Table 1:** Chemical compositions of cement [wt.%].

Cement Type	CaO	SiO_2_	Al_2_O_3_	Fe_2_O_3_	MgO	Na_2_O	K_2_O	SO_3_	TiO_2_	Cl
CEM I 42.5R	63.4	21.7	6.2	3.1	1.0	0.16	0.64	3.9	0.25	0.06

**Table 2:** Physical properties of cement.

Cement Type	Setting Start Time [min]	Setting End Time [min]	Compressive Strength [MPa]		Blaine Fineness [cm^2^/g]	Loss of Roasting [%]	Water Demand [%]
			After 2 Days	After 28 Days			
CEM I 42.5R	155	195	30.2	57.3	3504	3.4	27.5

**Table 3:** Physical properties of the aggregates [48,49,50].

Property	NA	GA	CA	AA
Water absorption WA_24_ (%)	0.6	15.2	17.3	14.8
Volume density *ρ*_a_ (kg/m^3^)	2620	380	850	1350
Open porosity P_o_ (%)	0.5	37	40	35
Bulk density in a loose state *ρ_b_* (kg/m^3^)	1740	200	610	850
Thermal conductivity of 40 cm layer of aggregate (W/m·K)	1.6	0.065	0.49	0.85

**Table 4:** Concrete mix proportion.

Components		Designation			
		TC NA	TC GA	TC CA	TC AA
		[kg/m^3^]			
CEM I 42.5R		500	700	700	700
Water		220	310	310	310
Aggregates	NA	1300	-	-	-
	GA	-	155	40	40
	CA	-	-	220	-
	AA	-	-	-	450
Superplasticizer		3.	4.9	4.9	4.9
Water/cement		0.44	0.44	0.44	0.44

**Table 5:** The summary of the size and quantity of the specimens for testing.

Test	Specimen Size [cm]	Quantity of the Specimens [Pieces]	Total Specimen Quantity [Pieces]
Volume density in a dry state	5 × 15 × 15	3	12
Compressive strength	15 × 15 × 15	6	24
Heat transfer–settings	30 × 30 × 5 (length × width × thickness)	2	8

**Table 6:** The obtained measurement results.

Components	Designation			
	TC NA	TC GA	TC CA	TC AA
*f _cm, cub_* [MPa]	67.5	45.8	57.3	62.8
*ρ* [kg/m^3^]	2244.4	1234.2	1529.8	1614.8
*λ* [W/m·K]	1.19	0.37	0.47	0.49
*δλ* [W/m·K]	0.044	0.014	0.017	0.019
*δλ* [%]	7.3	7.1	6.9	6.4
*R* [m^2^·K/W]	0.042	0.135	0.106	0.102

Fine aggregate: NA—natural, GA—foamed glass, CA—expanded clay, AA—fly ash.

## Data Availability

Data is contained within the article.

## Nomenclature

*A*	Surface area of specimen (m^2^)
*M*	Specimen mass (kg)
$\dot{Q}$	Heat flow rate (W)
*q*	Heat flux (W/m^2^)
*T*	Temperature (°C, K)
*δ*	Thickness of specimen (m)
*λ*	Thermal conductivity (W/m K)
*ρ*	Density (kg/m^3^)
